# Exploratory Study on Visual Acuity and Patient-Perceived Visual Function in Patients with Subretinal Drusenoid Deposits

**DOI:** 10.3390/jcm9092832

**Published:** 2020-09-01

**Authors:** Manjot K. Grewal, Shruti Chandra, Sarega Gurudas, Alan Bird, Glen Jeffery, Sobha Sivaprasad

**Affiliations:** 1 Institute of Ophthalmology, University College London, London EC1V9EL, UK; m.grewal@ucl.ac.uk (M.K.G.); shruti.chandra.18@ucl.ac.uk (S.C.); sarega.gurudas.17@ucl.ac.uk (S.G.); alan.bird@ucl.ac.uk (A.B.); g.jeffery@ucl.ac.uk (G.J.); 2 NIHR Moorfields Biomedical Research Centre, Moorfields Eye Hospital, London EC1V 2PD, UK

**Keywords:** subretinal drusenoid deposits, intermediate age related macular degeneration, retinal ageing, low-luminance questionnaire, low-luminance visual acuity, low-luminance deficit

## Abstract

Purpose: To investigate the value of visual acuity and patient-perceived visual function test when subretinal drusenoid deposits (SDD) are incorporated into the classification of age-related macular degeneration (AMD). A total of 50 participants were recruited into the study in these groups: healthy ageing (n = 11), intermediate AMD (iAMD) with no SDD (n = 17), iAMD with SDD (n = 11) and non-foveal atrophic AMD (n = 11) confirmed by two retinal imaging modalities. Best-corrected visual acuity (BCVA) and low luminance visual acuity (LLVA) were measured and low luminance deficit (LLD) was calculated. Participants were also interviewed with the low luminance questionnaire (LLQ). Linear regression was used to assess function–function relations. Compared with healthy participants, BCVA and LLVA scores were significantly reduced in the atrophic AMD group (*p* < 0.0001 and *p* = 0.00016, respectively) and in patients with SDD (*p* = 0.028 and *p* = 0.045, respectively). Participants with atrophy also had reduced BCVA (*p* = 0.001) and LLVA (*p* = 0.009) compared with the iAMD no SDD group. However, there were no differences in visual function tests between healthy aging and iAMD without SDD and between iAMD with SDD and atrophic AMD groups. The LLD score did not differ between groups. BCVA and LLVA correlated well. The LLQ did not correlate with visual function tests. This study shows that LLD is not a marker of disease severity as assessed clinically. Although LLQ is a good marker for disease severity using the current AMD classification, it does not differentiate between eyes with and without SDD. Eyes with non-macular geographic atrophy and SDD had lower function than eyes with no SDD and healthy controls.

## 1. Introduction

Visual impairment due to advanced age-related macular degeneration (AMD) is a global public health burden, with an estimated 196 million people being affected world-wide [1]. The global prevalence of early/intermediate AMD is approximately 8% [1]. Several investigators are currently evaluating various options to prevent progression to advanced AMD. The typical inclusion criteria for these clinical trials are people with intermediate AMD characterised by the presence of large drusen >125 μm and/or pigmentary abnormalities.

Recently, subretinal drusenoid deposits (SDD), otherwise termed reticular pseudodrusen (RPD), have been shown to co-exist with drusen in some eyes with early or intermediate AMD and these eyes are at higher risk of progression to advanced AMD [2,3]. Unlike drusen, which are located between the retinal pigment epithelium (RPE) and Bruch’s membrane, SDD are located internal to the RPE and are therefore in closer proximity to the photoreceptors when compared with drusen [4]. These SDDs have not been included in AMD classifications to date, although they have been found to have a profound correlation with rod recovery time [5,6]. It is, therefore, not clear whether or not eyes with intermediate AMD should be stratified into those with and without SDD to better define the impact of interventions in this condition.

Several investigators have evaluated visual function tests in healthy ageing and AMD based on existing classification. However, there is limited literature on the correlation of SDD with visual function. In particular, differences in visual function between healthy ageing, eyes with intermediate AMD with and without SDD and eyes that have progressed to non-foveal atrophy have not been explored. Insights from such evaluations are critical for defining inclusion criteria for future preventive options.

The standard test of visual function is the high luminance and high contrast best corrected visual acuity (BCVA), which represents a pure foveal cone function test. This test is not usually affected in early/intermediate AMD. However, when BCVA is assessed in low luminance (defined as low luminance visual acuity; LLVA), the visual function deficits are reported to be more pronounced and may even precede a decrease in BCVA [7,8,9].

The low-luminance deficit (LLD), or difference between LLVA and BCVA, has also been reported to be a predictive sign of disease progression and visual function losses [7].

In any clinical trial, objective functional measures should ideally correlate with subjective changes in visual function. Although most patients with early/intermediate AMD are asymptomatic, some complain of difficulty in performing their activities in varying illumination and at night [10,11,12]. These symptomatic patients may also be at higher risk of developing geographic atrophy, choroidal neovascularization (CNV) and three or more lines of visual acuity loss [13]. The low luminance questionnaire (LLQ) has been validated previously and could be used as patient-perceived visual outcome in AMD preventive trials [11]. However, the correlation of the measured visual acuity and subjective assessment of low luminance using the LLQ in healthy aging and intermediate AMD with and without SDD also has not been fully investigated.

The aim of this exploratory study was to assess the value of routinely performed visual function tests, BCVA, LLVA, and LLD, and subjective assessment by LLQ when SDD is introduced into the assessment of AMD severity and classification.

## 2. Experimental Section

The functional measures analysed are from the baseline data of a longitudinal study of visual function and structure done in Moorfields Eye Hospital from May 2017 to 2020. The study was approved by the Camden and Kings Cross NRES Committee London REC 16/LO/1317. Written informed consent was obtained from all participants and the study followed the tenets of the Declaration of Helsinki. 

### 2.1. Participants

The participants in this study had to have BCVA better than 50 ETDRS letters and were grouped according to the fundus characteristics of the study eye:

(1) No AMD or presence of druplets
(2) Intermediate AMD with no SDD (iAMD with no SDD)
(3) Intermediate AMD with at least 5 SDD (SDD group)
(4) Non-foveal atrophic AMD with intermediate AMD

Intermediate AMD was defined as having at least one large drusen (>125 µm), with or without pigmentary abnormalities. Diagnosis of these categories was based on at least two imaging methods done after mydriasis that included infrared reflectance, autofluorescence and spectral domain optical coherence tomography (SD-OCT) on Spectralis OCT2 (Heidelberg Engineering, Heidelberg, Germany); and color fundus photography (CFP) of the macula (Topcon; Tokyo, Japan).

#### 2.1.1. Exclusion Criteria

Participants were excluded if there was co-existent ocular disease (neovascular AMD, glaucoma or diabetic retinopathy, substantial cataract) in the study eye, significant systemic disease or history of medication known to affect visual function, epilepsy, history of major ocular surgery in the last 3 months or anticipated within the next 6 months following enrolment in the study eye and any allergies to adhesives or any other component used.

#### 2.1.2. Visual Function Tests

Participants first underwent a refraction protocol, followed by visual acuity measurement. BCVA was measured using a standard Early Treatment in Diabetic Retinopathy Study (ETDRS) for each eye. LLVA was measured by placing a 2.0 log neutral density filter over the eye and having participants read the same chart, with the aim of the filter to lower background luminance by 100-fold. These tests were performed monocularly with alternating charts for each eye at a 4 m distance. The difference between BCVA and LLVA in ETDRS letters was defined as the LLD score. The scores for the study eye were used in the analysis.

#### 2.1.3. Subjective Test

The LLQ is a 32-item questionnaire with six subscales related to low luminance settings: (1) extreme lighting, (2) mobility, (3) general dim lighting, (4) peripheral vision, (5) driving and (6) emotional distress [11]. Each question is scored on a scale ranging from 0, or maximal difficulty, to 100, or no difficulty. The questions are categorized into different subscales and averaged to generate one score per subscale. The weighted subscales are then averaged to produce a composite LLQ score.

#### 2.1.4. Statistical Analysis

Descriptive statistics were performed to assess baseline demographic variables for the AMD groups and controls using the chi squared test for categorical data or ANOVA/Kruskal Wallis test for continuous data. Applying the Shapiro-Wilk testing and using normal Q-Q plots revealed that the data were not normally distributed within each severity grade and therefore, non-parametric tests were used for functional parameters analysis. The relationship of BCVA and LLVA was assessed in each disease group. Pairwise differences of each AMD group were calculated using the nonparametric Kruskal Wallis test followed by post hoc uncorrected Dunn’s test.

The LLQ scores in each group were also compared. The nonparametric Kruskal Wallis test was carried out followed by Dunn’s uncorrected test for multiple comparisons to compare healthy ageing, intermediate AMD with and without SDD and those with atrophy on their composite and subscale scores on the LLQ. In each group, separate univariate linear regression models against the LLQ composite score were performed with BCVA, LLVA and LLD. Due to the exploratory nature of this study and small sample size, *p*-values were not adjusted for multiple comparisons. Inter-rater agreement for patient classification was assessed using Cohen’s kappa coefficient. Statistical analyses were performed using the statistical software SPSS (SPSS Statistics 23.0, SPSS Statistics for Windows, R2011; IBM Corp., Armonk, NY, USA) *p* < 0.05 was considered statistically significant. 

## 3. Results

### 3.1. Demographic and Clinical Characteristics of the Participants

A total of 50 participants were recruited; healthy ageing group (n = 11), iAMD no SDD (n = 17), iAMD with SDD (n = 11) and non-foveal atrophic AMD (n = 11). The mean age (±SD) of the cohort was 69.3 (±7.6) years and there were more female participants (60%) than male (40%). The mean age of the healthy aging group, iAMD with no SDD, iAMD with SDD and non-foveal atrophic AMD were 65.1 (±6.2) years, 66.3 (±8.1) years, 74.2 (±5.6) years and 73 (±6.0) years, respectively. The inter-rater agreement was high (Cohen’s kappa coefficient = 0.96) for evaluation and grading of colour fundus images for AMD classification.

### 3.2. Age-Related Decline in Visual Function

The visual function outcomes were not age-adjusted as SDD and atrophic changes are associated with advancing disease and linear regression showed no significant relationship between age in the healthy aging group and study-eye best-corrected visual acuity (*p* = 0.3170), low luminance acuity (*p* = 0.1115), low luminance deficit (*p* = 0.6165) and low luminance questionnaire (*p* = 0.7925).

### 3.3. Visual Acuity Assessments

Table 1 shows that there was a statistical difference between groups in BCVA and LLVA. However, the LLD was very similar between groups. Pairwise comparison showed there were no differences in either BCVA or LLVA between the healthy aging group and iAMD without SDD. BCVA and LLVA scores were significantly reduced in the non-foveal atrophic AMD group compared with the healthy group (*p* < 0.0001 and *p* = 0.0016, respectively). Patients with atrophy also exhibited poorer BCVA (*p* = 0.001) and LLVA (*p* = 0.009) scores compared with patients with intermediate disease without SDD. In patients with SDD, BCVA and LLVA, scores were lower compared with healthy participants (*p* = 0.028 and *p* = 0.045, respectively). There was no statistically significant difference between iAMD with SDD and non-foveal atrophic AMD.

**Figure 1:**
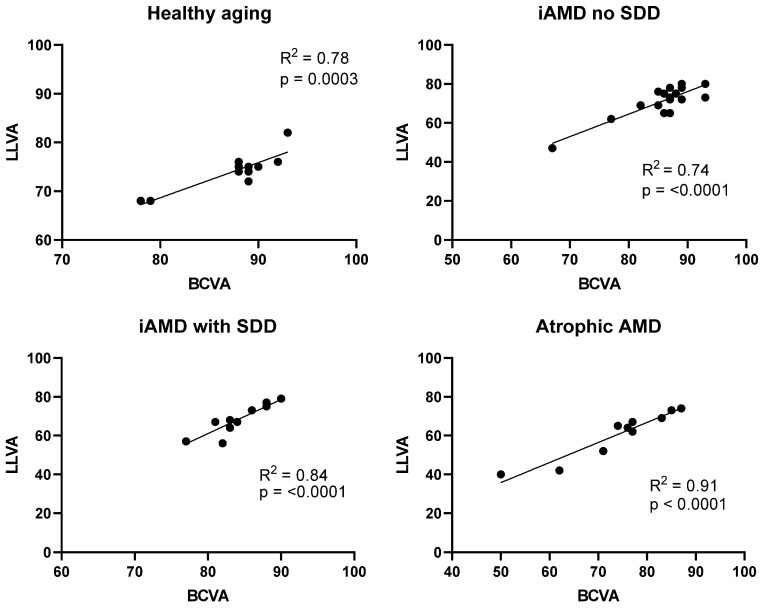
Scatter plots showing significant relationships between best-corrected visual acuity (BCVA) and low luminance visual acuity (LLVA) in healthy aging (n = 11, R^2^ = 0.78, *p* = 0.0003), iAMD no SDD (n = 17, R^2^ = 0.74, *p* < 0.0001), iAMD with SDD (n = 11, R^2^ = 0.84, *p* < 0.0001) and atrophic AMD (n = 10, one subject was excluded due to a high discrepancy between BCVA and LLVA, R^2^ = 0.91, *p* < 0.0001). iAMD = intermediate AMD, subretinal drusenoid deposits = SDD.

To understand the relationship between BCVA and LLVA, linear regression analysis of BCVA was performed against LLVA. There was a strong linear association between the two; BCVA was a significant predictor of LLVA in all disease groups, as shown in Figure 1. According to the R^2^ parameter, BCVA was able to explain 78% (*p* = 0.0003), 74% (*p* < 0.0001), 84% (*p* < 0.0001) and 91% (*p* < 0.0001) of the variance in LLVA for the healthy aging, iAMD no SDD, iAMD with SDD and atrophic AMD groups, respectively.

### 3.4. Subjective Assessment

**Figure 2:**
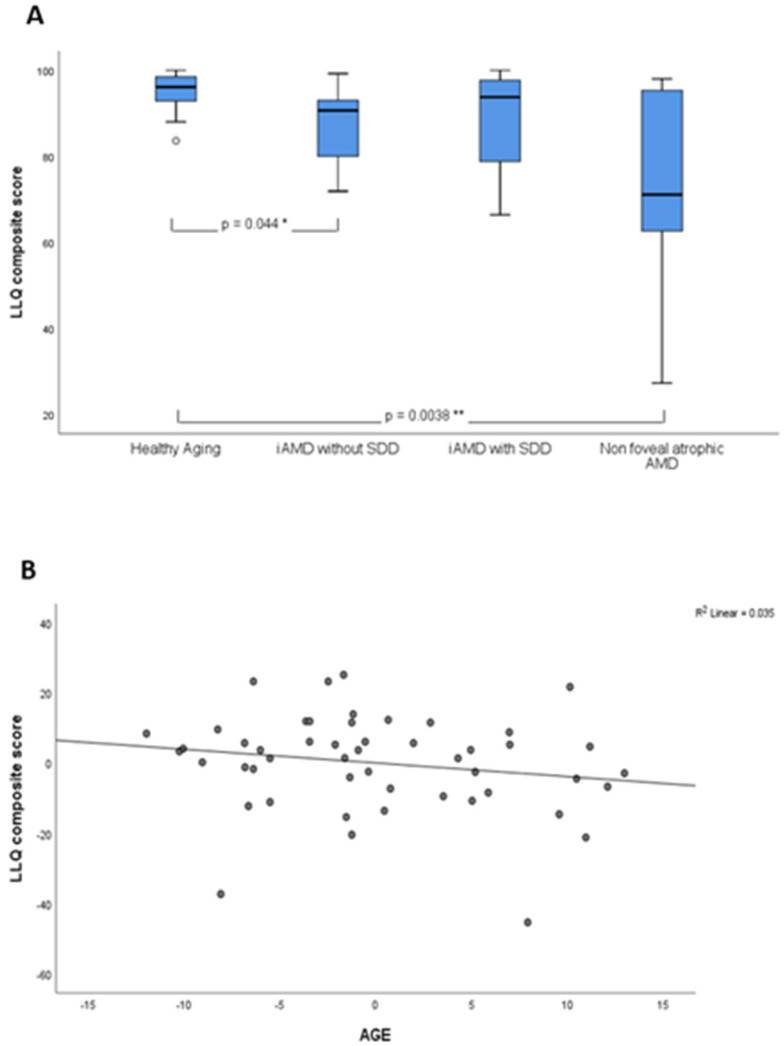
Low luminance questionnaire (LLQ) composite score shown as a function of disease and age. (**A**) Box plots showing LLQ composite score by disease group. Healthy aging (n = 11), iAMD without SDD (n = 17), iAMD with SDD (n = 11) and late AMD (n = 11). LLQ scores were statistically lower for the atrophic AMD group compared to healthy participants (*p* = 0.0038) and modestly reduced between iAMD without SDD and healthy participants (*p* = 0.044). (**B**) Linear regression showing the relationship of age against LLQ composite score in the full cohort after adjusting for disease (β coefficient = −0.390, R^2^ = 0.035, *p* = 0.211).

There was considerable variability in LLQ composite scores in eyes with non-foveal atrophic AMD compared with other groups, with participants obtaining very high scores (maximum 98.0), whereas moderate variability was observed in iAMD groups with or without SDD, as illustrated in Figure 2A. LLQ composite scores were statistically different in non-foveal atrophic AMD and healthy aging (*p* = 0.0038) and modestly different between iAMD without SDD and healthy aging (*p* = 0.044).

Linear regression analysis revealed a statistically significant association between LLQ composite score and age of the full cohort (*p* = 0.038), however it only explained 8.6% of the variance. This was, however, statistically non-significant when adjusting for disease status (β coefficient = −0.390, R^2^ = 0.035, *p* = 0.211), as shown in Figure 2B.

There was significant differences in the LLQ subscale scores between groups as shown in Table 2. Patients with non-foveal atrophic AMD had consistently lower median subscale scores compared with other groups. The subscale scores were significantly lower for patients with atrophy when compared with the healthy aging group for extreme lighting (*p* = 0.0017), emotional distress (*p* = 0.0034) and overall LLQ composite score (*p* = 0.0038). The scores were also lower for the iAMD without SDD compared with healthy participants for extreme lighting, emotional distress and composite score (*p* = 0.030, *p* = 0.047 and *p* = 0.044, respectively).

Univariate linear regression of functional outcomes against LLQ composite score is shown in Table 3. There was no significant association between LLQ composite score and any functional outcomes in healthy aging, iAMD without SDD and atrophic AMD participants. LLQ composite score was weakly associated with BCVA (*p* = 0.047) in patients with SDD.

## 4. Discussion

This study evaluated the correlation of visual acuity tests and patient-perceived visual function in healthy ageing and in different severity groups of AMD. We found no significant difference in BCVA or LLVA between iAMD with no SDD and healthy ageing. However, BCVA and LLVA were reduced significantly in eyes with iAMD with SDD compared to healthy ageing. This discrimination is important as it stratifies iAMD into those with and without functional loss based on the presence or absence of SDD.

Previous reports have reported reduced LLVA in AMD patients with drusen >125 µm compared with healthy participants [8,9,10]. However, these studies used clinical severity scales of AMD based on traditional classification, which does not include the presence of SDD as a distinct entity. In this study, we categorized eyes with SDD as a separate cohort and found that the reduced LLVA in iAMD eyes may indeed be driven by the subgroup of patients with SDD.

On further comparison, eyes with non-foveal atrophy were found to have significantly worse BCVA and LLVA when compared with controls (healthy ageing) and eyes with iAMD without SDD. Interestingly, the iAMD group with SDD had similar functional outcomes as eyes with non-foveal atrophy, i.e., BCVA as well as LLVA were reduced in both groups. Previous studies by Sunness et al. have shown LLVA to be reduced in eyes with non-foveal geographic atrophy (GA) and also predict subsequent vision loss [7,14]. However, we found that the presence of SDD results in functional outcomes similar to that of non-foveal atrophic AMD, suggesting that eyes with SDD is a marker of advanced disease, even though no structural changes of atrophy are visible on clinical examination or imaging. However, SDD may disturb overlaying photoreceptors, leading to outer segment shortening, inner segment deflection and eventual loss along with outer retinal layer gaps over large deposits, which are abnormalities commonly observed in histology and imaging around and ahead of GA progression [15,16]. Specifically, in eyes with GA, shortening of outer segments and photoreceptor loss are seen distant from the GA [15].

These SDD go through a lifecycle of changes, finally resulting in regression [4]. Eyes with regression of SDD develop outer retinal atrophy associated with underlying choroidal thinning [16]. This form of atrophy represents a late form of AMD and is structurally distinct from geographic atrophy (GA) in terms of loss of RPE in the latter [16]. Studies have also shown preferential rod dysfunction in patients with SDD [3,5,6,17]. We hypothesize that functionally, eyes with SDD regression behave in an analogous manner as eyes with GA, consequently explaining the congruity in the BCVA and LLVA outcomes in these groups. Therefore, our study shows that the presence of SDD is an indicator of severe disease and the functional outcomes are as poor as those with non-foveal atrophy.

There is discordance in literature regarding LLD measure. Our study found no statistically significant difference in LLD between groups, which contrasts with results from Puell et al. who found significant difference in LLD in the non-advanced AMD groups [8]. Wu et al. noted a difference in LLD between participants with non-foveal GA and control group only, but no significant difference in LLD between control and in the non-atrophic AMD groups [9]. However, Cocce and colleagues (2018) reported no mean difference in LLD between groups in their cohort [10]. This may be due to the fact that we divided our groups to include SDD as a separate group and the baseline visual acuity was good across all groups in our study. The heterogeneity in outcomes indicates that retinal function changes may be independent of the currently used clinical grading scales based on structure. This finding may in part be influenced by the presence of SDD, which is not currently included in the classification [9,18].

LLVA is a measure of mesopic function, predominantly cone-mediated in reduced illumination [8,14,19]. We found a strong correlation between BCVA and LLVA across all groups and a likely explanation for this would be that both tests are inherently dependent on foveal cone function, consequently resulting in a similar functional mechanism. Stockman and Sharpe explained that the visual acuity in a mesopic setting requires integrated cone function mediated by post-receptoral pathways and a disruption of this would lead to a drop in LLVA, worsening the LLD [20]. A similar reduction in both BCVA and LLVA could be secondary to mechanical disruption and disorientation of photoreceptors, thereby reducing spatial resolution at both illuminance levels [21,22,23]. As LLD is a difference of BCVA and LLVA, any improvement or worsening in LLD has to be interpreted in the context of actual values of BCVA and LLVA [14]. For example, an improved LLD might be a result of worsening foveal photopic vision, thereby lowering the BCVA, but LLVA may not be affected to the same degree as parafoveal cones are less responsive to changes in illumination than foveal cones [14]. Similarly, a parallel reduction in BCVA and LLVA could result in a non-significant difference in LLD, which probably explains the indiscriminate LLD result between groups. In our study, LLD ranged from 13.5 to 16.3 letters, a difference of 2.8 letters between groups, which is clinically not meaningful when visual acuity variability ranges from 5 to 10 letters [24,25]. Therefore, our study suggests that LLD is not a biomarker of increasing disease severity.

We also evaluated whether LLQ composite scores deteriorate with age and disease severity. Although our results showed a decrease in LLQ composite score with age, this was insignificant when adjusted for disease severity. These results substantiate previous findings that age is not associated with LLQ [26,27].

However, LLQ composite score was found to be an accurate marker for disease severity, although it could not differentiate between iAMD with and without SDD. The subscales that were most affected were extreme lighting and emotional distress in iAMD no SDD and the non-foveal atrophy group. The subscale on difficulty in dim lighting did not reach statistical significance. Interestingly, there was no difference in composite score between healthy controls and iAMD with SDD. This is difficult to interpret but it may be that eyes with SDD eyes are not affected by extreme lighting. There were large standard deviations in LLQ subscales in this small sample sized study, which could confound our findings.

Univariate linear regression analysis showed no association between LLQ and LLVA and LLD.

As LLQ was designed specifically to assess patient-perceived difficulty in low luminance, it is more likely to elicit rod function, whilst LLVA is predominantly a cone-mediated test. It is reasonable to expect discordance between these two parameters [11]. Overall, no significant association was found between BCVA and LLVA against LLQ. This is substantiated by another study where no significant associations were found between BCVA, LLVA and LLQ in traditional visual acuity measurement with ETDRS charts at 4 m [27]. However, Thompson et al. found a significant relationship between computerized LLVA and LLD against the LLQ [27]. Previous studies that have shown a good correlation of LLD with subjective assessment assessed only the question specific to night vision. Given the small sample size and variability within each AMD group, our sample size did not permit such a specific analysis [9]. However, we believe that multiple visual function tests are required to encapsulate the extent of the LLQ questionnaire as some activities may involve more than just a rod mediated response.

The limitations in this study include the small sample size and the lack of SDD-only group, with the latter being very challenging to recruit. However, given that the primary aim of this study was to examine the value of visual function measures and the degree of self-reported difficulties in low luminance conditions when subretinal drusenoid deposits (SDD) are incorporated into the clinical AMD classification, the lack of an SSD-only group does not detract from our interpretation of the results.

Despite the small sample size in each group, the disease was very well characterised, with SDD confirmed by at least two imaging techniques. There was high inter-rater agreement for patient classification (Cohen’s kappa coefficient = 0.96). In addition, all assessments were highly standardized and carried out by a single observer, limiting interobserver variability, indicating the validity of our findings for the cohort we examined, and can be extrapolated with caution. Although current findings cannot be directly applied in clinical practice due to the small sample size, these findings would be useful for designing future clinical trial endpoints. We also cannot rule out that some eyes in the iAMD with no SDD and non-foveal atrophy had regressed SDD. A surrogate for regressed SDD is to measure the thickness of outer nuclear layer, however this is challenging to measure due to the undulations caused by the presence of large drusen.

## 5. Conclusions

In conclusion, our study suggests that LLD is not a good marker of increasing disease severity. Our findings validate that LLVA is predominantly a foveal function test and accordingly, BCVA and LLVA show good correlation where the decrease in function is maximally seen in eyes with non-foveal atrophy. The LLQ that mainly assesses the subjective integrity of rod function did not correlate with visual acuity parameters, which are cone-mediated tests. Our study results suggest the need to re-classify the AMD severity scale by incorporating SDD based on visual function tests. However, this postulation needs to be validated by investigating the structural–function correlation in eyes with SDD in terms of the quantity and area covered by SDD. We also recommend that intervention trials designed to decrease progression to advanced AMD exclude eyes with SDD and non-foveal atrophy or take these characteristics into account when drawing conclusions.

## Figures and Tables

**Table 1:** Cross-sectional analysis at baseline for all functional outcome measures for all groups.

	Group, Mean ± SD, Median (Minimum, Maximum)				Pairwise Comparisons Post Hoc Uncorrected Dunn’s Test *p* Value						
Test	Healthy Aging (n = 11)	iAMD No SDD (n = 17)	iAMD with SDD (n = 11)	Non-Foveal Atrophic AMD (n = 10) †	Overall *p* Value *	Healthy Aging vs. iAMD No SDD	Healthy Aging vs. iAMD with SDD	Healthy Aging vs. Non-Foveal Atrophic AMD	iAMD No SDD vs. iAMD with SDD	iAMD No SDD vs. Non-Foveal Atrophic AMD	iAMD with SDD vs. Non-Foveal Atrophic AMD
**BCVA (letters)**	87.6 (±4.8)	85.7 (±6.1)	83.6 (±4.3)	74.2 (±11.2)	0.0005	0.237	0.028	<0.0001	0.218	0.001	0.065
	78, 89, 93	67, 87, 93	77, 83, 90	50, 76.5, 87							
**LLVA (letters)**	74.1 (±3.89)	71.1 (±8.2)	67.3 (±8.2)	60.8 (±12.1)	0.0077	0.385	0.045	0.0016	0.181	0.009	0.230
	68, 75, 82	47, 73, 80	56, 67, 79	40, 64.5, 74							
**LLD (letters)**	13.5 (±2.3)	14.6 (±4.3)	16.3 (±4.6)	13.4 (±3.7)	0.4244	-	-	-	-	-	-
	10, 14, 17	9, 14, 22	11, 15, 26	9, 12.5, 20							

* Overall *p* value from non-parametric Kruskal Wallis test, † One participant was excluded in the non-foveal atrophic AMD group as the LLVA score was an outlier. SDD: subretinal drusenoid deposits; AMD: age-related macular degeneration; BCVA: best-corrected visual acuity; LLVA: low luminance visual acuity; LLD: low luminance deficit; iAMD: intermediate AMD.

**Table 2:** The LLQ subscale and composite scores between each subject group.

	Group, Mean ± SD, Median (Minimum, Maximum)				Pairwise Comparisons Post Hoc Uncorrected Dunn’s Test *p* Value †						
LLQ Categories	Healthy Aging (n = 11)	iAMD No SDD (n = 17)	iAMD with SDD (n = 11)	Atrophic AMD (n = 11)	Overall *p*-Value *	Healthy Aging vs. iAMD No SDD	Healthy Aging vs. iAMD with SDD	Healthy Aging vs. Non-Foveal Atrophic AMD	iAMD No SDD vs. iAMD with SDD	iAMD No SDD vs. Non-Foveal Atrophic AMD	iAMD with SDD vs. Non-Foveal Atrophic AMD
**Extreme lighting**	90.5 ± 9.0, 93.8	77.5 ± 13.1, 75.0	81.3 ± 15.4, 78.1	61.8 ± 26.9, 68.8	0.015	0.030	0.210	0.0017	0.432	0.197	0.060
	(78.1, 100.0)	(53.1, 100.0)	(53.1, 100.0)	(21.9, 95.8)							
**Mobility**	97.0 ± 4.6, 100.0	93.6 ± 7.1, 95.8	92.4 ± 10.7, 100.0	86.0 ± 18.0, 91.7	0.451	-	-	-	-	-	-
	(87.5, 100.0)	(79.2, 100.0)	(75.0, 100.0)	(45.8, 100.0)							
**General dim lighting**	95.5 ± 6.6, 100.0	89.5 ± 10.7, 91.7	93.2 ± 9.9, 100.0	76.1 ± 23.0, 79.2	0.051	-	-	-	-	-	-
	(83.3, 100.0)	(66.7, 100.0)	(75.0, 100.0)	(41.7, 100.0)							
**Peripheral vision**	96.2 ± 8.6, 100.0	94.1 ± 8.7, 100.0	96.2 ± 8.6, 100.0	80.3 ± 26.7, 91.7	0.092	-	-	-	-	-	-
	(75.0, 100.0)	(75.0, 100.0)	(75.0. 100.0)	(25.0, 100.0)							
**Driving**	93.0 ± 10.3, 95.0	83.5 ± 17.0, 90.0	79.5 ± 29.7, 90.0	58.6 ± 36.5, 50.0	0.157	-	-	-	-	-	-
	(65.0, 100.0)	(45.0, 100.0)	(5.0, 100.0)	(0.0, 100.0)							
**Emotional distress**	100.0 ± 0.0, 100.0	92.7 ± 10.4, 100.0	91.5 ± 15.4, 100.0	75.6 ± 29.5, 93.8	0.032	0.047	0.100	0.0034	0.861	0.216	0.200
	(100.0, 100.0)	(68.8, 100.0)	(50.0, 100.0)	(25.0, 100.0)							
**Composite**	94.8 ± 5.2, 96.1	87.3 ± 9.1, 90.6	87.7 ± 12.9, 93.8	72.8 ± 24.6, 71.1	0.032	0.044	0.203	0.0038	0.543	0.240	0.106
	(83.6, 100.0)	(71.9, 99.2)	(66.4, 100.0)	(27.3, 98.0)							

* *p*-values specified relate to the significance in mean rank difference between study groups (non-parametric Kruskal Wallis test); † Post hoc pairwise comparisons for subscales with overall *p*-value < 0.05.

**Table 3:** Univariate linear regression of functional tests against LLQ Composite Score in all AMD severity groups.

Outcome Measure	Healthy Aging			AMD No SDD			AMD with SDD			Non-Foveal Atrophic AMD		
	B Coefficient	Standardized Coefficient	*p* Value	B Coefficient	Standardized Coefficient	*p* Value	B Coefficient	Standardized Coefficient	*p* Value	B Coefficient	Standardized Coefficient	*p* Value
BCVA	−0.299	−0.276	0.411	0.551	0.368	0.146	1.828	0.608	0.047	0.076	0.041	0.910
LLVA	−0.203	−0.153	0.654	0.186	0.166	0.523	0.927	0.592	0.055	0.239	0.141	0.697
LLD	−0.734	−0.321	0.337	0.444	0.208	0.424	−1.359	−0.489	0.127	−1.859	−0.337	0.341

One participant was excluded in the non-foveal atrophic AMD group due to an unusually high LLD score.
